# Association of *Schistosoma haematobium* infection morbidity and severity on co-infections in pre-school age children living in a rural endemic area in Zimbabwe

**DOI:** 10.1186/s12889-020-09634-0

**Published:** 2020-10-19

**Authors:** Tariro L. Mduluza-Jokonya, Thajasvarie Naicker, Luxwell Jokonya, Herald Midzi, Arthur Vengesai, Maritha Kasambala, Emilia Choto, Simbarashe Rusakaniko, Elopy Sibanda, Francisca Mutapi, Takafira Mduluza

**Affiliations:** 1 Optics & Imaging, Doris Duke Medical Research Institute, College of Health Sciences, University of KwaZulu-Natal, Durban, KwaZulu-Natal South Africa; 2 Department of Biochemistry, University of Zimbabwe, P.O. Box MP 167, Mt Pleasant, Harare, Zimbabwe; 3 Department of Surgery, College of Health Sciences, University of Zimbabwe, P.O. Box MP 167, Mt Pleasant, Harare, Zimbabwe; 4 Department of Community Medicine, College of Health Sciences, University of Zimbabwe, P.O. Box MP 167, Mt Pleasant, Harare, Zimbabwe; 5 Twin Palms Medical Centre, 113 Kwame Nkrumah Avenue, Harare, Zimbabwe; 6 Institute for Immunology and Infection Research and Centre for Immunity, Infection and Evolution, School of Biological Sciences, University of Edinburgh, Ashworth Laboratories, King’s Buildings, Charlotte Auerbach Rd., Edinburgh, EH9 3JT UK

**Keywords:** Schistosomiasis, Under-5 mortality rate, Communicable diseases, Acute respiratory infection, Fever of unknown origin, Pre-school age children, Pneumonia, Dermatophytosis

## Abstract

**Background:**

Individuals living in *Schistosoma haematobium* endemic areas are often at risk of having other communicable diseases simultaneously. This usually creates diagnostic difficulties leading to misdiagnosis and overlooking of schistosomiasis infection. In this study we investigated the prevalence and severity of coinfections in pre-school age children and further investigated associations between *S. haematobium* prevalence and under 5 mortality.

**Methods:**

A community based cross-sectional survey was conducted in Shamva District, Zimbabwe. Using random selection, 465 preschool age children (1–5 years of age) were enrolled through clinical examination by two independent clinicians for the following top morbidity causing conditions: respiratory tract infections, dermatophytosis, malaria and fever of unknown origin. The conditions and their severe sequels were diagnosed as per approved WHO standards. *S. haematobium* infection was diagnosed by urine filtration and the children were screened for conditions common in the study area which included HIV, tuberculosis, malnutrition and typhoid. Data was analysed using univariate and multinomial regression analysis and relative risk (RR) calculated.

**Results:**

Prevalence of *S. haematobium* was 35% (145). The clinical conditions assessed had the following prevalence in the study population: upper respiratory tract infection 40% (229), fever of unknown origin 45% (189), dermatophytosis 18% and malaria 18% (75). The odds of co-infections observed with *S. haematobium* infection were: upper respiratory tract infection aOR = 1.22 (95% CI 0.80 to 1.87), dermatophytosis aOR = 4.79 (95% CI 2.78 to 8.25), fever of unknown origin aOR = 10.63 (95% CI 6.48–17.45) and malaria aOR = 0.91 (95% CI 0.51 to1.58). Effect of schistosomiasis coinfection on disease progression based on the odds of the diseases progressing to severe sequalae were: Severe pneumonia aOR = 8.41 (95% CI 3.09–22.93), *p* < 0.0001, complicated malaria aOR = 7.09 (95% CI 1.51–33.39), *p* = 0.02, severe dermatophytosis aOR = 20.3 (95% CI 4.78–83.20):*p* = 0.03, and fever of unknown origin aOR = 1.62 (95%CI 1.56–4.73), *p* = 0.02.

**Conclusion:**

This study revealed an association between schistosomiasis and the comorbidity conditions of URTI, dermatophytosis, malaria and FUO in PSAC living in a schistosomiasis endemic area. A possible detrimental effect where coinfection led to severe sequels of the comorbidity conditions was demonstrated. Appropriate clinical diagnostic methods are required to identify associated infectious diseases and initiate early treatment of schistosomiasis and co-infections in PSAC.

## Background

Human schistosomiasis is a parasitic disease caused by blood flukes called trematode worms of the genus *Schistosoma*. Schistosomiasis predominantly affects people in low- and middle-income countries [1], where there is poor provision of water and sanitation. The mainstay of morbidity control is currently through provision of mass drug administration (MDA) to target age groups. However, children under 5 years old are usually left out of the MDA exercise due to lack of paediatric size medicine. From the 78 countries affected by schistosomiasis about 206.4 million people required treatment [2, 3]. In sub-Saharan Africa alone, about 52 endemic countries reported moderate to high prevalence of schistosomiasis infection [4]. Individuals living in schistosomiasis-endemic areas are often at risk to several pathogens simultaneously [5]. These coinfections could occur by chance or through host exposure to other locally endemic disease agents [6]. Alternatively, schistosomiasis may increase or decrease the risk for another infection [7]. Studies on co-infection in adults have shown that schistosomiasis co-infection hinders diagnosis and treatment of other communicable diseases [8, 9]. To date, however, little focus has been given to pre-school age children (PSAC), who were hitherto regarded as a low risk group. However, recent studies suggest that they may have similar risk to adults [10]. The associations of schistosomiasis to co-infections in PSAC has not yet been fully described.

The World Health Organization (WHO) Sustainable Developmental Goal (SDG) 3 aims to reduce under-5 mortality to at least as low as 25 per 1000 live births in every country by 2030 [4]. In Zimbabwe, the top causes of morbidity and mortality in PSAC include acute respiratory tract infections (ARI), malaria, diarrhoea, fever and skin diseases [11, 12]. In this study we investigated the prevalence and extent of morbidity associated with *S. haematobium* coinfections in children under the age of 5 years in a *Schistosoma* endemic district of Zimbabwe. Furthermore we investigated the relationship between the selected condition’s severity and under-5 mortality rate.

## Methods

### Study site and design

In this community cross sectional survey, we recruited children from 19 out of the 22 different villages of Shamva district. Recruitment was conducted during their expanded immunisation programme (EPI) gatherings and at rural health centres. The study was carried out in Shamva district which is in Mashonaland Central province, Zimbabwe [12]. Shamva recorded the highest prevalence of schistosomiasis in Zimbabwe at 62.3%, during schistosomiasis and soil transmitted helminths (STH) national mapping exercise [13]. The district lies 945 m above sea level, has a warm climate and average temperature of 20.2 °C and an annual rainfall of 887 mm [14]. Shamva district has high farming activity due to presence of fertile soil. Residents get their water supply from Mazowe river which spans through the district [12].

### Study inclusion criteria

Participants recruited into the study were lifelong residents of the Shamva district. The PSAC were aged between 1 to 5 years and had a previously reported inclusion criteria [14]. In addition participants had to have a Mantoux test reaction < 5 mm and a normal nutrition status (based on clinical examination, which included mid upper arm circumference measurement and weight-for-age as well as height-for-age measurements).

### Sample size

Participant selection was by simple random sampling. Mothers were requested to bring their children to the clinic or EPI meeting points. The required sample size was calculated to be 363 participants using the Dobson formula [13], where the known schistosomiasis prevalence in Shamva district was 62% [13, 15].

### Ethical statement

Ethical approval was obtained from Medical Research Council of Zimbabwe (MRCZ/A/2435). Approval was also obtained from the Provincial and District Medical Directors and Community Leaders. Written informed consent was obtained from the parents or guardians of the children. All participants with confirmed infections were offered appropriate treatment.

### Data collection

A questionnaire designed for the study (supplementary file 1) was administered to the caregivers/parents and medical record of each child was accessed for those who had been hospitalised over the past year. The coinfections were selected from top causes of morbidity in Zimbabwean children under-5 years of age [15]. Information was extracted from the Zimbabwe demographic health survey, and Zimbabwe provincial and district census statistics [12, 14–21]. The data captured on under-5 mortality was then compared with the prevalence of the top mortality conditions in children which included HIV, malaria, diarrhoea and schistosomiasis [22–25].

### Clinical examinations

**Fig. 1:**
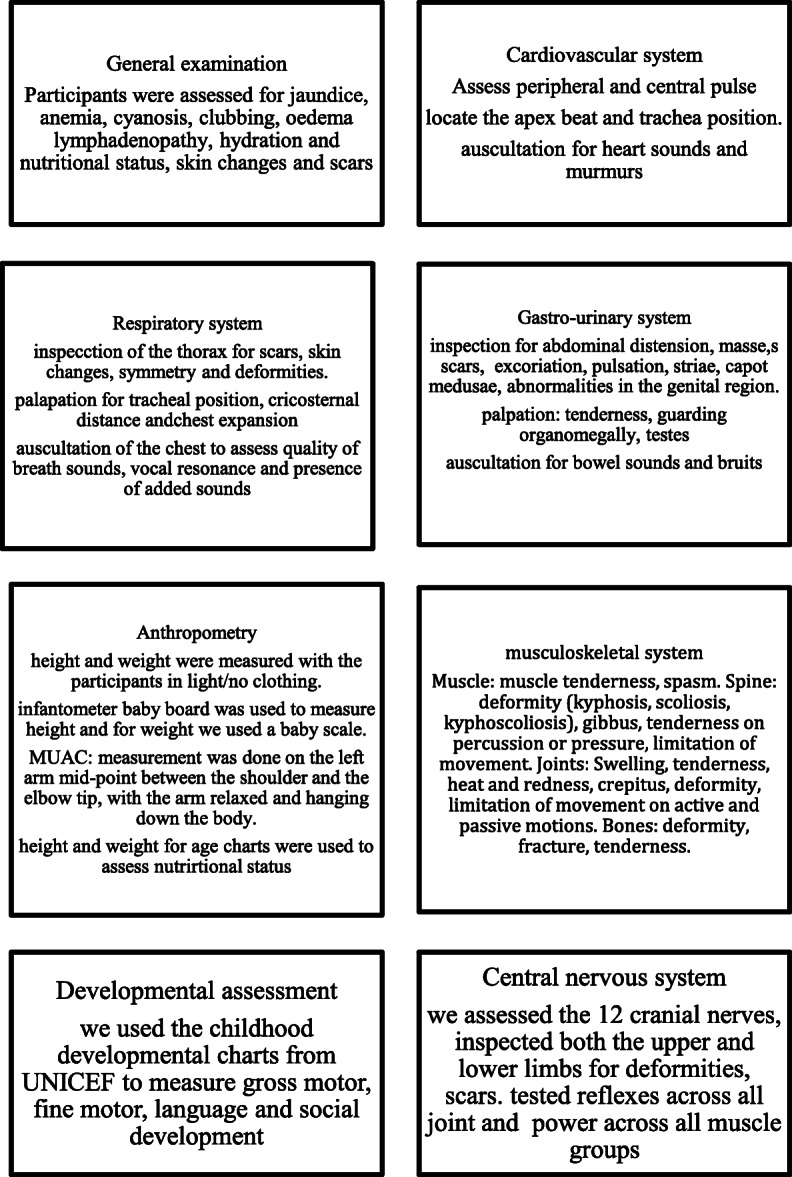
Clinical examinations protocol listing the details of physical examinations conducted on each participant as adopted from standard clinical practices [26, 27]

The clinical examinations were conducted at a health facility or at EPI allocated facilities on PSAC (*n* = 415) by **two** medical practitioners independent of each other. The examinations were conducted based on a protocol as shown in Fig. 1.

### *S. haematobium* infection diagnosis

Urine samples were collected using a wide open container that made it easier for the child to urinate in, children 1 year and below used paediatric urine collector attached by a clinician. The caregivers then brought the samples to the team that were examined as follows:

### Haematuria check and parasitology

Haematuria check and parasitology was done as previously described [14]. The parasitology team recorded the results independently of the clinical team.

### Blood processing and analysis

Serum and plasma obtained from each child was processed and tested for Toxoplasmosis, rubella, cytomegalovirus, herpes simplex virus 1 and 2, HIV, typhoid and Hepatitis. The sera was processed using the Maglumi 4000 chemiluminescence immunoassay analyser (CLIA). Children diagnosed to have infection were managed appropriately by the doctors in the study and the community nurse.

### Co-infections diagnosis

#### Upper respiratory tract infection (URTI)

URTI was diagnosed on clinical signs and symptoms after excluding allergy and influenza and as per IMCI guidelines [28–31]. Assessment of URTI progression into severe sequel (severe pneumonia was defined as per WHO guidelines [32, 33].

#### Fever of unknown origin (FUO)

FUO was defined as children who within the past 6 months had been admitted with a temperature of 38.5 °C and no other diagnosis found after blood, urine and stool cultures as represented from their medical records [34]. Assessment of FUO progression into severe sequel (seizures) were described as change in movement, attention or loss of consciousness in a child diagnosed with FUO, without a history of neurological symptoms [35].

### Malaria

#### Parasitology examination

A few drops of anticoagulated participants blood specimen with EDTA were used for parasite identification and count. Briefly, about two drops of the blood sample were collected on glass slide for preparation of thin and thick blood smears in duplicate. The smears were stained with 10% Geimsa working solution for 10 mins, thin smears were fixed in 100% methanol before Geimsa staining. Malarial parasites were identified under a microscope and parasite load was calculated after counting asexual parasites per 200 white blood cells using the formula assuming that the mean WBC in children 1–5 years old is 11,000/μL [36].

Parasite count/ *μ*L = number of observed asexual parasite × 11,000 WCC/ *μ*L all divided by 200 WCC [37]. Assessment of malaria progression into severe sequel or complicated malaria was described as per WHO guideline for severe malaria: hyperparasitemia (parasite load > 100,000 parasites/*μ*L), persistent vomiting, respiratory distress, convulsion (more than two in 24 h), posturing, comma, discoloration of urine, unable to walk, sit, and stand or unable to feed and drink in infants, hyperpyrexia and hypoglycaemia [33, 38].

#### Dermatophytosis

Skin scrapings were collected from children with signs of dermatophytosis, examined by microscopy on a warmed potassium hydroxide treated slide [39]. Assessment of dermatophytosis disease progression into severe sequel or severe dermatophytosis was described as ringworms covering greater than 20% surface area using the paediatric burns chart [40].

### Statistical methods

Initial analysis was to determine a relationship between the top clinical conditions which children below 60 months old presented with at health facilities and the *S. haematobium* infection status. Data analysis was performed by STATA version 15, using regression and descriptive analysis. Results were reported as adjusted odds ratios (aORs) with 95% confidence interval (CI), along with the test for significance, as previously described [41]. A relationship was determined of being *S. haematobium* infected and the clinical conditions advancing to the severe sequels, this was done by multinomial regression analysis adjusted for sex, age and *S. haematobium* infection which gave adjusted odds ratio. Infection intensity for *S. haematobium* was defined as previously described that is arithmetic average egg count per ten millilitres of at least two urine samples.

## Results

**Fig. 2:**
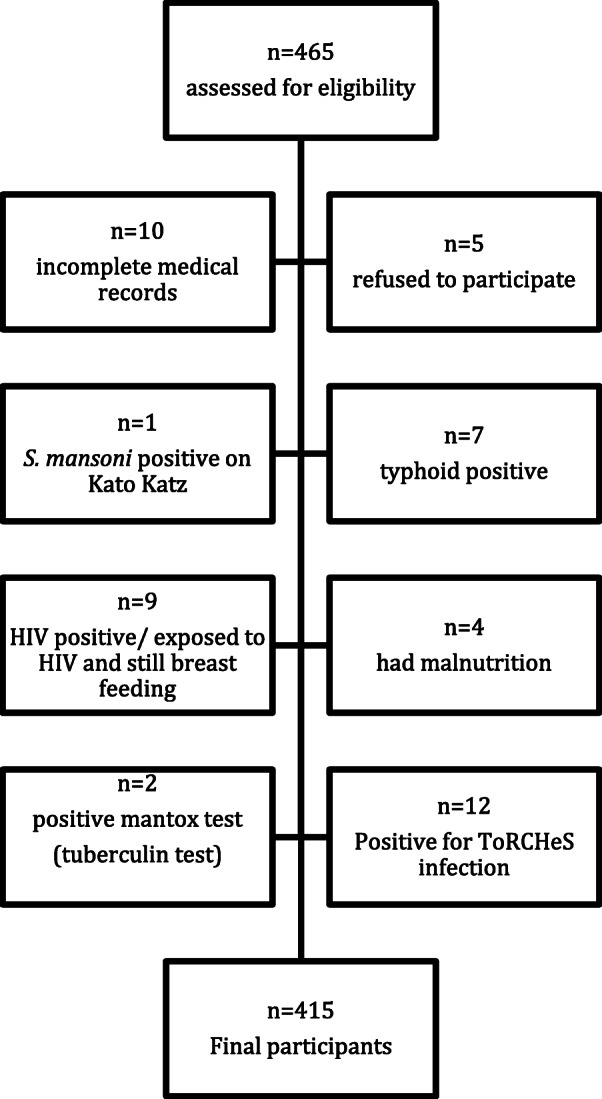
Screening profile showing the participants who were excluded from the study and the type of conditions they had at time of recruitment

Screening involved 465 PSAC, aged 1 to 5 years from the Shamva district, 415 met the eligibility criteria and consented to be part of the study (Fig. 2). Participants has an equal sex ration and the mean age was 3.4 years. Relative risk (RR) based on schistosomiasis and sex was 0.93 (95% CI 0.76–1.14) with *p* = 0.48, based on age it was also statistically insignificant. Age was shown to increase the risk of *S. haematobium* infection; as the children grew older (Table 1). Children suffering from malaria had *P. falciparum* as the parasite.

**Table 1:** Relative risk of *S. haematobium* associated with participants demographic characteristics

Characteristics		Relative Risk	95%CI	*p*-value
Sex		0.93	0.76 to 1.14	0.48
Age (years)	1	0.47	0.08 to 2.38	0.91
	2	0.92	0.67 to 1.27	0.62
	3	0.73	0.52 to 1.02	0.07
	4	1.12	0.91 to 1.56	0.21
	5	1.35	0.90 to 2.04	0.15

### Infection prevalence in the study population

The prevalence of *S. haematobium* as measured by urine filtration was 35.1% (145), While among the study participants, 40% (229) presented with URTI, 45% (188) with FUO and 18% (75) with dermatophytosis and 18% with malaria (Table 2). The prevalence of co-infections with *S. haematobium* was: URTI 35% (80), malaria 33.3% (22), FUO 55% (91) and dermatophytosis 67% (40).

**Table 2:** Clinical conditions among children aged 1–5 years in a schistosomiasis-endemic district of Zimbabwe

Clinical Conditions		Schistosomiasis infection Status		Total prevalence of the condition in the study population (N = 415)
		Negative (***N*** = 270)	Positive (***N*** = 145)	
Upper Respiratory Tract Infection (URTI)	Negative	121	65	40%
	Positive	149	80	
Dermatophytosis	Negative	244	95	18%
	Positive	25	50	
Fever of Unknown Origin (FOU)	Negative	172	54	45%
	Positive	97	91	
Malaria	Negative	220	120	18%
	Positive	50	25	

### Regression analysis of the co-infections

In univariate analysis, the following associations with schistosomiasis infection had significant odds ratio (OR): URTI OR = 1.98 (95% CI 1.657–2.48), dermatophytosis OR = 5.10 (95% CI 2.99–8.72) and FUO OR = 9.07 (95% CI 5.70–14.44) (Table 3). In multivariable analysis, after adjusting for age, sex and infection status, the following were independently associated indicating the following adjusted odds ratio (aOR): dermatophytosis aOR = 4.79 (95% CI 2.78–8.25) and FUO aOR = 10.63 (95% CI 6.48–17.45).

**Table 3:** Crude and adjusted odds ratio of the association between Schistosomiasis infection and other clinical conditions

Other Clinical Conditions	Crude Odds Ratio	Adjusted Odds Ratio	Relative risk
Upper Respiratory Tract Infection	**1.98**^a^ **(1.66–2.48)**	1.22 (0.80–1.87)	1.01 (0.84–1.2)
Dermatophytosis	**5.10** ^a^**(2.99–8.716)**	**4.79**^a^ **(2.78–8.252)**	**1.38**^a^**(1.22–1.56)**
Fever of Unknown origin	**9.07** ^a^**(5.70–14.44)**	**10.63**^a^ **(6.48–17.45)**	**2.38**^a^ **(1.90–2.983**
Malaria	0.91 (0.54–1.54)	0.91(95% CI 0.51 to 1.58)	0.98 (0.90–1.08)

^a^significant at 5% level of significance

### Association between Under-5 mortality rate and Schistosomiasis in Zimbabwe

**Fig. 3:**
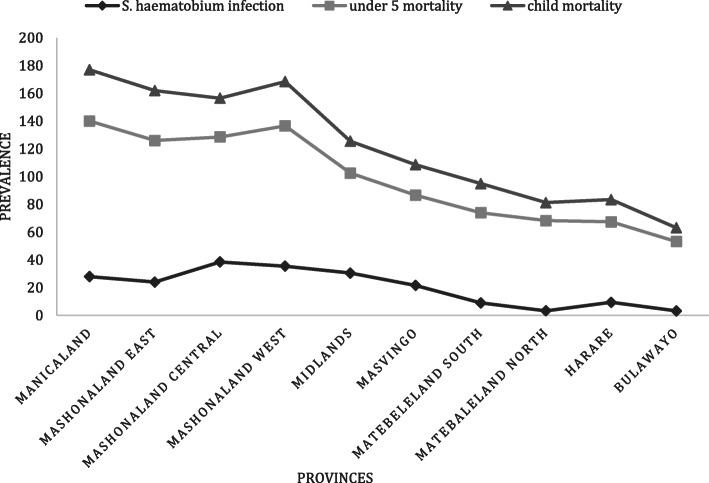
Trends showing schistosomiasis prevalence in comparison to child and under 5 mortality per 1000 live births from Zimbabwe provinces. The data was extracted from the national demographic and health survey, Zimbabwe and Zimbabwe provincial and district census statistics [12, 14–21]

There was a positive relationship between *S. haematobium* infection with child mortality and under-5 mortality in Zimbabwean Provinces (Fig. 3). Provinces with an increased schistosomiasis prevalence (Manicaland, Mashonaland East, West and Central) all showed an increased under-five child mortality rate. Whereas, Matabeleland North and Bulawayo had both a decrease in *S. haematobium* infection prevalence and mortality ratios.

### Effects of Schistosomiasis co-infections on severity of disease progression

In multivariate analysis after adjusting for sex, age and *S. haematobium* infection, PSAC with schistosomiasis and URTI had an eight-fold higher odds of developing severe pneumonia than children with URTI alone aOR = 7.90 (95% CI 2.76–27.5), *p* = 0.008 (Table 4). Children with schistosomiasis and malaria had a 7-times greater odds of developing complicated malaria aOR = 7.09 (95% CI 1.51–33.39), *p* = 0.005. Schistosome-infected children with dermatophytosis had a 20-fold higher odds of progressing to severe dermatophytosis aOR = 20.3 (95% CI 4.78–83.2; p = < 0.001) compared with children who did not have schistosomiasis. Among children with FUO, those who also had schistosomiasis coinfection had twice the chance of seizures aOR = 1.62 (95% CI 1.56–4.73).

**Table 4:** Odds ratio of having severe sequelae from co-infections with schistosomiasis in PSAC from an endemic district

Co-infected with schistosomiasis	Sequelae experienced	aOR	95% Confidence interval
Upper Respiratory Tract Infections	Severe Pneumonia	**8.41**^a^	3.09 to 22.93
Malaria	Severe malaria	**7.09**^a^	1.51 to 33.39
Dermatophytosis	Severe dermatophytosis	**20.3**^a^	4.78 to 83.2
Fever of Unknown Origin	Seizures	**1.62**^a^	1.56 to 4.73

^a^significant at 5% level of significance

Children with URTI and *S. haematobium* infection had a 650% higher risk of progression to severe pneumonia compared to those without coinfection, relative risk (RR) = 7.5 (95%CI 2.92–19.23), *p* < 0.0001. Coinfection with *S. haematobium* increased the risk of children with dermatophytosis progressing to severe and persistent dermatophytosis by 750% (RR = 8.5 (95%CI 1.2–60.27), *p* = 0.03; risk of having seizure following FUO diagnosis in *S. haematobium* coinfected individuals was 132% higher than in those without co-infection (RR = 2.32 (95%CI 1.12–4.80), *p* = 0.02 and *S. haematobium* infected children who acquired malaria as a coinfection had a 1200% higher risk of the disease progressing to complicated malaria RR = 12 (95%CI 11.53–94.53), p = 0.02 compared to the children without coinfection.

## Discussion

Children growing up in resource limited rural areas have high exposure to schistosomiasis infection and a high likelihood of associated coinfections with diseases prevalent in these communities. A strong positive association between schistosomiasis and URTI; dermatophytosis and FUO was observed. The trends revealed an association between schistosomiasis and under-5 mortality rate from the national data provided by the Ministry of Health in Zimbabwe. There was a negative association between schistosomiasis and malaria, though participants with the two as co-infections had a greater likelihood of presenting with complicated malaria. Similarly, participants with URTI, FOU and dermatophytosis as coinfections had a higher likelihood of having severe sequelae of the diseases.

A positive association of schistosomiasis prevalence in under-5 and child mortality rate in the district mirrored the trends in different provinces of Zimbabwe. The provinces that had a high schistosomiasis rate also had high mortality rate [11, 15]. This made us wonder if schistosomiasis co-infections was possibly worsening the disease courses as we also found that in co-infections there was a greater chance of the diseases running a severe course [15]. It is necessary to explore the effects of schistosomiasis and other diseases co-infections in all the top morbidity and mortality causes in PSAC. Early schistosomiasis treatment in PSAC has the potential to lower the under-five mortality rate, by reducing the incidence of severe sequelae of the top morbidity conditions, which are also top causes for mortality in this age group. The development of a co-infection algorithm advocating for intensified screening for co-infections should be shared with policy makers in low and middle income countries.

*S.haematobium* infection was shown to increase with age, this is in keeping with other studies done [42–46]. This is probably due to the fact that the older children do more activities in contaminated water like swimming and house chores unlike the younger PSAC who are only exposed during bathing. In most cases caregivers know to boil the water before bathing the infants which decreases the chance of acquiring S. haematobium infection. Focus has to be made to decrease exposure of the older PSAC to contaminated water. This might need to include tackling schistosomiasis from the snail host stage.

Children infected with *S. haematobium* had a 20-fold higher odds of severe dermatophytosis, after adjusting for other clinical conditions. *S. haematobium* infection had a 38% increased risk of getting dermatophytosis. There is no previous documentation between *S. haematobium* and dermatophytosis. In literature, extensive dermatophytosis was noted in a case reports involving *S. mansoni* [47, 48]. It is postulated that *S. mansoni* exacerbated dermatophytosis by lowering immunity due to the liver involvement or by suppression of the T- helper 1 system which is involved in suppressing fungal infections [49, 50]. Dermatophytosis cause great morbidity in PSAC in Zimbabwe [11]. Dermatophytosis, when severe is associated with psychological trauma via discrimination. Clinicians in schistosomiasis endemic areas need to be aware of this condition in order to decrease the morbidity associated with dermatophytosis. Further studies on schistosomiasis association with dermatophytosis and immunological profiling are recommended.

Children with schistosomiasis associated with plasmodium malaria coinfection had reduced risk. However, malaria infection has been documented to be exacerbated/ameliorated by schistosomiasis co-infection [18–20]. The enhanced T-helper 1 cellular activity and an increase in anti-interferon-gamma cytokine production causes a protective effect against the malarial parasite [51]. Of note is that although schistosomiasis was demonstrated to have a protective effect on malaria, in the event of co-infection; malaria infection had 7-fold chance of exacerbating to complicated malaria requiring hospital admission in the PSAC. This finding makes it crucial for PSAC to be included in national schistosomiasis control programs, to reduce or eliminate morbidity and mortality in children in this age group. However, the conundrum in PSAC coinfections and schistosomiasis require urgent attention, as this is the age of growth and development during which the immune system is developing; and when childhood vaccines are delivered. Further studies are required to understand co-infection in PSAC inclusive of malaria infection outcome.

There was a 10-fold chance of finding FUO and schistosomiasis as a coinfection, children with schistosomiasis had 138% risk of FUO. The proportion of patients discharged with undiagnosed FUO remain unchanged even though there has been enormous development in medicine over time [52]. The cause of febrile illness is not identified in approximately 9–51% of patients, this is even higher in resource limited areas endemic for childhood illnesses [53]. Children who had both FOU and schistosomiasis had a 2-fold chance of having serious sequelae such as seizures. Most clinician tend to think of other conditions in contrast to neglected tropical diseases when patients present with a fever [33]. It might be necessary to make it a priority to screen for schistosomiasis when a child from an endemic area presents with a fever. However, further immunological investigations are necessary in-order to find out if the fever is due to schistosomiasis infection or exacerbation of a co-infection, during the early immunological responses to diseases manifestation.

In our study the odds of having URTI was 2% higher in schistosomiasis infected children with a 1% increased risk of *S. haematobium* infected children acquiring URTI. Furthermore, in the coinfected cohort odds of ending up with severe pneumonia was 7-fold compared to schistosomiasis negative population. This is a very significant finding as acute respiratory infections (ARIs) are the leading cause of morbidity and mortality in children under the age of five in Zimbabwe [11]. Thus tackling schistosomiasis crisis in this age group will have enormous contribution in reducing the childhood mortality and morbidity in schistosomiasis endemic areas.

The strength of this study revealed the major morbidity and mortality causes in PSAC from our study area which also fit into most of the low income countries were schistosomiasis is endemic. The main limitation of this study is there is a possibility that the children may have had more than two co-infections which might make our data biased, however we did thorough clinical examinations and laboratory tests in all the participants to rule this out. We also considered socioeconomic status as a confounding factor by focusing on children with a similar background and in the same villages with similar lifestyles. Given the likelihood of co-infections, children who present with schistosomiasis should be screened for other conditions.

## Conclusions

This study revealed an association between schistosomiasis and top morbidity conditions of URTI, dermatophytosis, malaria and FUO in PSAC living in a schistosomiasis endemic area. The study also showed that there is need for an appropriate clinical diagnostic method to identify associated infectious diseases and initiate early treatment of schistosomiasis and co-infections in PSAC. Furthermore, we demonstrated that coinfection led to severe sequelae of the clinical conditions, that have a high impact on morbidity and mortality in children under the age of five. This brings out the importance of including PSAC in national schistosomiasis control programs and the development of appropriate diagnostic tools.

## Supplementary information

**Additional file 1.** Study questionaire.

## Data Availability

The statistical data on the parasitology and clinical scores used to support the findings of this study are available from the corresponding author upon request.

## Abbreviations

aOR: Adjusted odds ratios
ARI: Acute respiratory tract infection
CI: Confidence interval
EPI: Expanded program on immunization
FUO: Fever of unknown origin
HIV: Human Immunodeficiency Virus
MDA: Mass Drug Administration
MRCZ: Medical Research Council of Zimbabwe
PSAC: Preschool age children
RR: Risk ratio/relative risk
SDG: Sustainable Development Goals
STH: Soil transmitted helminths
ToRCHeS: Toxoplasmosis, rubella, cytomegalovirus, hepatitis and syphilis
URTI: Upper respiratory tract infection
WBC: White blood cells
WCC: White cell counts
WHO: World Health Organization
