# Supplementary material for: Association of Schistosoma haematobium infection morbidity and severity on co-infections in pre-school age children living in a rural endemic area in Zimbabwe
Source: BMC Public Health. 2020 Oct 19;20:1570. doi: 10.1186/s12889-020-09634-0 (PMC7574170; doi:10.1186/s12889-020-09634-0)
Supplement: Supplementary file 1 — Additional file 1. Study questionaire. [file 12889_2020_9634_MOESM1_ESM.docx]

**Questionnaire**

**USaCuF: SCHISTOSOMIASIS IN CHILDREN UNDER THE AGE OF 5**

**This questionnaire is 4 pages long- please complete ALL pages.**

## Questionnaire to be completed at the time of visit with parents and enrollment of children into the study and only after obtaining parents consent and assent of participating child.

Date __________________

Ref number ______________________

Village ______________________________

| SEX | F | M |
| --- | --- | --- |
| AGE |  | |
| HISTORIAN |  | |
| PRIMARY CAREGIVER |  | |

**SECTION A**

1. Has the child ever been treated for bilharzia?

YES/NO

1. Has anyone from the child’s family ever been treated for bilharzia

YES/NO

1. What is the domestic water source *(circle as many as appropriate)*
2. unprotected well
3. River
4. Dam
5. Upgraded well
6. Borehole
7. Tap
8. Other (specify)
9. Where does the child normally go to the toilet? *(circle as many as appropriate)*
10. Bush
11. cat sanitation
12. Latrine/toilet
13. Other (specify)
14. Is there a latrine at your home? *(circle either yes or no*)

Yes No

1. If yes, are there any problems in using it? *(circle either yes or no*)

Yes No

If yes, explain ____________________________________________________

## Did your child do any of these activities in the water the past week? (circle as many as appropriate; if activity circled, ask other questions in table for each separate contact)

|  | How many times? | Where? | (If River or Dam) | At what time? (e.g. 0800, 1600) |
| --- | --- | --- | --- | --- |
|  |  | River/Dam/Well/ Other | name of place or nearest village/school |  |
| Swimming |  |  |  |  |
| Playing in the water |  |  |  |  |
| Bathing |  |  |  |  |
| Laundry |  |  |  |  |
| Washing dishes |  |  |  |  |
| Washing (face and legs) |  |  |  |  |
| Collecting water |  |  |  |  |
| Fishing |  |  |  |  |
| Crossing river |  |  |  |  |
| Other_______________ |  |  |  |  |

1. How many times has your child been to the river or dam in the last week? _______
2. Where does the child normally get treated when unwell? (Circle as many as appropriate)
3. At home using herbal medicines
4. At home using western medicine
5. At the traditional healer
6. At the local clinic
7. Other please specify__________________________________________

**SECTION B**

1. Nutrition ________ breastfeeding Y/N

If no when was baby weaned___________________

1. Immunisations

| BCG | MEASLES | DTP BOOSTER |
| --- | --- | --- |
| PENTAVALENT 1 | PENTAVALENT 2 | PENTAVALENT 3 |
| OPV 1 | OPV 2 | OPV 3 |
| OPV 4 |  |  |
| ROTAVIRUS 1 | ROTAVIRUS 2 | ROTAVIRUS 3 |
| PNEUMOCOCCAL 1 | PNEUMOCOCCAL 2 | PNEUMOCOCCAL 3 |

1. Developmental mile stones -__________________________________________________
2. HIV status ___________________________________
3. Past medical history

____________________________________________________________________________________________________________________________________________________________________________________________________________________________________________________________________________________________________________________________________________________

1. Family history

|  |
| --- |

1. Family medical history

| asthma | atopy |
| --- | --- |
| Other |  |

1. Systems Review

General

| Wellbeing | Appetite |
| --- | --- |
| Weight changes | energy |
| Excessive sleep | Lacking sleep |

Respiratory and cardiovascular

| Shortness of breath | coughing |
| --- | --- |
| Excessive sweating on breastfeeding | Easy tiring on breastfeeding/playing |
| Turning dusky blue | Lower limb swelling |

Gastrointestinal

| Mouth sores | Dental problems |
| --- | --- |
| vomiting | diarrhea |
| constipation | stomachache |

Urogenital

| ____________nappy changes a day | heamaturia |
| --- | --- |
| Straw colored urine |  |

Nervous System

| Clumsiness when walking | Clumsiness when handling thimgs |
| --- | --- |
| Concerntration problems | faints |
| fits |  |

**SECTION C**

1. Physical examination

ANTHROPROMETRIC MEASUREMENTS

| MUAC |  |
| --- | --- |
| Head Circumference |  |
| Weight |  |
| Height |  |
| Weight for age |  |
| Height for age |  |
| Weight for Height |  |

GENERAL

| Jaundice | Pallor | Cyanosis |
| --- | --- | --- |
| Clubbing | Edema | Lymphadenopathy |
| Respiratory rate ________ | Heart rate __________ |  |

CHEST

|  |
| --- |
|  |
|  |
|  |

CARDIOVASCULAR

|  |
| --- |
|  |
|  |
|  |

GASTROINTESTINAL

|  |
| --- |
|  |
|  |
|  |

CENTRAL NERVOUS SYSTEM

|  |
| --- |
|  |
|  |
|  |

MUSCULOSKELETAL SYSTEM

|  |
| --- |
|  |
|  |
